# Safeguarding natural ecosystems can protect population health: advancing approaches to bridge the health–ecology divide

**DOI:** 10.1016/j.lanplh.2025.101377

**Published:** 2025-11-05

**Authors:** Peninah Murage, Charlotte Hicks, Valerie Kapos, Santhuri Naidoo, Syreen Hassan, Sarah Whitmee

**Affiliations:** Centre on Climate Change and Planetary Health, Department of Public Health, Environment and Society, London School of Hygiene & Tropical Medicine, London, UK; United Nations Environment Programme World Conservation Monitoring Centre (UNEP-WCMC), Cambridge, UK; United Nations Environment Programme World Conservation Monitoring Centre (UNEP-WCMC), Cambridge, UK; United Nations Environment Programme World Conservation Monitoring Centre (UNEP-WCMC), Cambridge, UK; Faculty of Epidemiology and Population Health, Department of Non-communicable Disease Epidemiology, London School of Hygiene & Tropical Medicine, London, UK; Centre on Climate Change and Planetary Health, Department of Population Health, London School of Hygiene & Tropical Medicine, London, UK

## Abstract

Human health stands to benefit from a deeper understanding of the mechanisms by which ecosystems affect health and wellbeing. However, achieving this understanding requires overcoming conceptual and practical challenges in both public health and ecology. Despite growing recognition of the importance of natural ecosystems for human health, the health sector has yet to fully integrate this ever-growing body of evidence to inform policy and practice. Substantial conceptual differences underpin the disciplinary divide between health and ecology. For example, environmental health research disciplines, such as epidemiology, emphasise the adverse effects of environmental exposures, such as water and air pollutants, as well as naturally occurring hazards, such as radon or arsenic. By contrast, ecology focuses on nature’s contributions to people through ecosystem services, including food provision, climate regulation, or disaster management. These conceptual differences create a misalignment in evidence generation, in setting priorities for policy, and in the implementation of solutions. Methodological differences further complicate the alignment of health and ecology datasets, particularly when exposures and outcomes occur across different spatiotemporal scales. The disciplines also differ on how to define pathways from exposure to health and how to quantify and communicate effects. Consequently, each discipline often reinforces its existing views instead of leveraging the combined knowledge base for a broader understanding. This Personal View outlines practical steps to bridging the divide and fostering transdisciplinary collaboration by recognising the dynamic interactions between health and natural ecosystems, integrating conceptual frameworks across disciplines, and addressing methodological challenges in assessing impacts.

## Integration of evidence on nature–health interactions into policy and practice in the health sector

The role of natural ecosystems in health is increasingly recognised; however, the health sector has been slow to generate and integrate emerging evidence into policy and practice. Global trends in human health indicators have improved substantially over the past century,^1^ underpinned by the supporting role of nature and natural ecosystems. These systems play a crucial role in providing essential resources such as food for people and animals, clean water, energy, medicines, and other natural materials.^2^ Functioning ecosystems also support human health and wellbeing by regulating the Earth’s natural systems and processes and buffering against natural disasters.^3^

Despite advances in establishing the role of natural ecosystems,^1^ public health research has yet to fully integrate ecological considerations into research and policy development and remains predominantly focused on a bio-medical approach to understanding and treating disease. Similarly, environmental sciences have not sufficiently captured the health and wellbeing implications of biodiversity and ecosystem services, including actions such as nature-based solutions (NbS), or expressed these influences in ways that are meaningful to public health. Integrative approaches such as NbS, One Health, and Planetary Health can bridge gaps within and between public health and environmental sectors to meet multiple, interlinked challenges (panel). However, without a strong consensus to address health challenges emerging from the loss of biodiversity and ecosystem services and without a robust evidence base to identify the most effective interventions, mobilising investment to support these integrative approaches remains challenging. Furthermore, many promising NbS have yet to be implemented at the scale necessary to realise their full potential.

Here, we examine the challenges that have hindered the transition from conceptual engagement to practical, on-the-ground collaboration between the two disciplines. We propose viable solutions that adapt existing conceptual and methodological frameworks to support the joint identification of research and monitoring priorities and inform the development of common metrics.

### Human health–ecosystem interactions are complex

To fully harness the potential of nature–health interactions, it is essential to promote health and wellbeing in parallel with ecosystem health. Public health can benefit from a more complete understanding of the complex interlinkages between natural ecosystems and human health and well-being. Health professionals should consider how these interactions can be positive, negative, or neutral and how they vary across different scales and contexts.

Failure to consider these dynamics poses a barrier to integrating the links between ecosystems and human health into policy and practice across both sectors. The field of ecology should recognise that characterising the health and wellbeing benefits of natural ecosystems can serve as a powerful lever to mobilise public and political support for conservation and can help to unlock investment in natural capital. This notion is particularly true in contexts in which NbS have been shown to support health outcomes and the broader Sustainable Development Goals in implementing communities.

The interlinkages between natural ecosystems and human health can be examined from several perspectives. For example, there are environmental determinants of illness mediated by ecosystems, including naturally occurring harmful substances such as arsenic, radon, lead, ultraviolet radiation, and disease vectors. Disciplines such as environmental epidemiology and exposure science are dedicated to studying exposure pathways, including air, food, water, and soil, and quantifying health burdens in terms of mortality, illnesses, or population attributable fractions. Public health risk assessments aim to identify hazards, evaluate risk, and formulate appropriate responses or actions to minimise exposure and reduce risk to populations.

Health outcomes are linked to biodiversity and ecosystem services—eg, access to urban green and blue spaces, which support both physical and mental health;^6^ the role of biodiversity in regulating the emergence and transmission of zoonotic disease;^7^ the provision of natural resources, such as water, food, and conventional and alternative medicines;^2^ and natural processes that contribute to regulating climate and mitigating natural disasters,^2^ thereby preventing injuries and loss of life.^3^ Degradation of natural ecosystems and loss of biodiversity can disrupt resource availability; degrade air, water, and soil quality; and interrupt ecological functions, thereby increasing the risk of emergence and transmission of infectious diseases.^7^ The potential for NbS to safeguard health and wellbeing is increasingly recognised;^8,9^ however, their effects on health outcomes remain under-researched.

Actions intended to improve health outcomes, including health promotion and clinical interventions, can inadvertently damage ecosystems and indirectly harm populations.^10,11^ The production, transportation, and storage of pharmaceuticals and single-use disposable medical supplies, for example, generate toxic waste and consume large amounts of water and natural resources.^10^ The global health-care sector accounts for up to 5% of total environmental impacts, such as air and noise pollution, and greenhouse gas emissions, with estimates varying across countries and according to indicators considered.^11^

## Challenges in jointly setting priorities for research and generating evidence

Differences in framing research questions and methodologies, such as those between epidemiology and ecosystem assessments, remain major obstacles to generating evidence that effectively articulates the interactions between ecosystems and health. Research in neither sector fully acknowledges the evidence produced by the other, and each discipline focuses on well-known examples that align with its internal narratives. For instance, health professionals often emphasise the effect of urban green spaces on mental health. In contrast, there is insufficient evidence on health impacts—both beneficial and adverse—of other ecosystems, such as mangroves, which are more extensively featured in the ecological literature. This section reflects on how disciplinary differences in research prioritisation, evidence generation, and evidence utilisation contribute to reinforcing the divide.

### Different disciplinary perspectives in defining pathways to health

Health and ecology are guided by differing priorities and approach evidence generation from different perspectives, creating challenges in aligning research objectives. To understand the influence of nature on human health and wellbeing, ecologists focus on the structure, functions, and services of natural ecosystems as well as the measures that can be implemented to protect or restore ecosystems. In contrast, public health professionals prioritise understanding environmental risk factors, disease causality, and actions to protect human health. Even within interdisciplinary teams, reaching consensus on which drivers of change, risk factors, outcomes, or actions to prioritise can be challenging. Furthermore, environmental change often affects health through complex and indirect pathways, frequently mediated by ecological processes. Consequently, in resource-limited and time-limited environments, research and policy have tended to favour simplified pathways. For example, the relationship between heat exposure and health outcomes is well established in the epidemiological literature.^12^ In contrast, pathways linking biodiversity change with the prevalence and influence of vector-borne diseases are difficult to capture and communicate owing to the complex and nuanced exposure–response relationships that vary across habitats, pathogens, hosts, and vector species.^7^

### Differing time and spatial scales in exposure and outcomes

Some environmental exposures and changes affect health over longer timeframes and broader spatial scales than those that are typically encountered in public health research. For example, the degradation of aquatic habitats, leading to the depletion of fish stocks and negative effects on nutrition, might take years to have substantial effects, and the associated health effects might occur at different spatial and temporal scales from the immediate effect on the habitat. In contrast, environmental risk factors, such as air and water pollutants, pose more immediate health risks and are therefore often prioritised in epidemiological studies. Approaches such as natural capital accounting provide a useful framework for understanding effects and opportunities. However, these assessments are typically conducted at the landscape level and encompass multiple stakeholder groups, making it challenging to disentangle the specific health and wellbeing benefits that flow to particular groups and thereby constraining targeted action.^13^

### Differences in methods to quantify and communicate outcomes

Disciplinary differences in estimating potential and actual exposures, risks, and consequences—both positive and negative—can hinder the generation and communication of evidence that resonates across stakeholder groups. In epidemiology, hazard and impact assessments, along with randomised controlled trials, are often used to clarify health effects and inform actions to treat and minimise risks. Ecology also involves impact assessments; however, risks and effects are more likely to be considered from the perspective of biodiversity and ecosystem services and are assessed over long time periods with high degrees of uncertainty. Furthermore, approaches such as natural capital accounting, which assign monetary and other types of value to nature, are almost exclusively applied in the environmental sector,^14^ with minimal input from the health sector.

## Towards transdisciplinarity

Drawing from existing best practices, we explore practical steps towards conceptual and methodological integration for aligning research and policy priorities.

### Conceptual integration

Conceptual frameworks are valuable tools for disentangling the complex relationships between natural ecosystems and human health. We highlight two frameworks commonly applied in public health and environmental sciences—the Driving Force-Pressure-State-Exposure-Effect-Action (DPSEEA) framework and the Intergovernmental Science-Policy Platform on Biodiversity and Ecosystem Services (IPBES) framework. Identifying points of intersection between these approaches is the first step to achieving greater integration between the two sectors. Such integration can facilitate a holistic assessment that simultaneously considers natural ecosystems alongside other drivers of human health and their effects.

The DPSEEA framework is widely applied in environmental health and epidemiological research, providing a structured approach to analysing the relationship between environmental hazards and health outcomes.^15^ The application of this framework has been centred on hazard assessments to identify causal pathways linking environmental hazards to health effects and on actions to prevent exposure or treat those affected. Although the framework recognises driving forces, pressures, and changes in the state of the environment, these dimensions are only broadly stated rather than quantitatively assessed in epidemiological studies. This limitation does not reflect a conceptual flaw in the framework but indicates the narrow scope of its application. DPSEEA is inherently flexible, and the revised eDPSEEA framework formally recognises the inter-connectedness between ecosystem services and human health.^16^

The IPBES framework similarly integrates drivers and effects, aiming to describe the components of social–ecological systems and their interactions.^17^ With its emphasis on biodiversity and nature’s contributions to people (ecosystem services), this framework is widely used in ecological or ecosystem assessments. Key strengths of the IPBES framework include its flexibility to accommodate complex drivers and impacts of biodiversity loss as well as its recognition of the need to incorporate indigenous and local knowledge systems. Some of the applications of the IPBES framework also explicitly integrate health dimensions, such as the recently approved IPBES Assessment Report on the Interlinkages Among Biodiversity, Water, Food and Health.^18^

These two frameworks are conceptually distinct—IPBES focuses on nature’s positive contributions to health, whereas DPSEEA primarily assesses the adverse health effects of environmental change. Mapping their synergies can help to align overlapping components for broader cross-sectoral applicability. For instance, both frameworks recognise the contribution of underlying social, cultural, political, and economic dimensions that provide opportunities for developing a shared theory of change. Assessing areas of discrepancy can further identify gaps in each framework that might be addressed by adapting elements from the other.

The Ecosystems Cascade Model^19^ offers a promising framework for integrating monitoring of actions and evaluation of outcomes. This model provides a useful starting point for bridging methodologies in health and ecology to achieve a meaningful integration. It examines how changes in components of natural ecosystems can trigger a cascading chain of events in other ecological processes and in societal aspects. This model also offers a systematic means of identifying and assessing pathways from ecosystem modifications to potential human health outcomes, accounting for intermediary ecological processes and environmental outcomes.^19^

To illustrate this approach, we have briefly adapted the framework, drawing from a study evaluating the heat adaptation effects of natural regeneration in landscapes using agroforestry techniques.^20^ In this practical example, native trees are integrated into croplands to promote species diversity, improve soil fertility, and enhance livelihoods in East African drylands. One outcome is the regulation of the local microclimate, which benefits outdoor agricultural workers by providing improved thermal comfort while they work under shade trees. The adapted Ecosystems Cascades Model (figure) summarises a multidisciplinary perspective on quantifying the cascading effect of actions (trees in croplands) on ecological functions, ecosystem services, environmental outcomes (mediated by ecosystems), and subsequent health and economic outcomes.

In the figure, the adapted Cascades Model is aligned with the DPSEEA framework to identify crucial opportunities for evaluating outcomes and tracking progress. Rigorous evaluation of effects and progress requires pooling expertise across disciplines, thereby supporting methodological alignment—eg, through the development of shared indicators and metrics that meet the needs of all stakeholders. Analysing the effects of actions is an integral component of both the DPSEAA and IPBES frameworks and can be applied to a wide range of actions, including policy implementation, public health initiatives, and ecosystem management activities.

### Integration of existing methodologies in health and ecology

Aligning methods for evidence generation can be achieved through several established approaches—eg, by defining shared objectives across disciplines to guide priorities for research, policy, and practice. Community-based and stakeholder-based approaches offer opportunities for developing joint win-win actions that deliver multiple benefits across both the environment and health sectors or no-regrets actions that avoid or minimise negative effects across both sectors.^21^ The Convention on Biological Diversity’s Global Action Plan on Biodiversity and Health provides an example of such an integration. This voluntary framework seeks to integrate biodiversity–health interlinkages into policies, strategies, and programmes at all levels.^22^ It emphasises cross-sectoral collaboration by engaging diverse stakeholders, including indigenous groups, women and youth, and advocates for the use of holistic approaches such as One Health, alongside the creation of systems to monitor progress, share best practices, and measure effectiveness.^22^

Another promising approach is developing joint metrics and indicators to clarify what, how, and when to assess drivers, exposures, outcomes, actions, and their effects. Metrics are quantifiable data points that measure a specific process or outcome, whereas indicators are a combination of metrics to provide a broader understanding and inform strategic action. To support integration, both metrics and indicators must be relevant across sectors. For example, where ecosystems are mediators of environmental exposures, joint indicators could be developed to quantify the thresholds at which environmental determinants become harmful for health. Indicators could also be designed to identify the quantity or quality of ecosystems required to eliminate or reduce risks without compromising ecological integrity or generating negative consequences for health.

Potential practical approaches include incorporating environmental factors into disease surveillance and using natural capital accounting alongside health impact assessments. One study quantified the role of urban forests in modifying air pollution concentrations and the potential to minimise illness and deaths attributed to poor air quality.^23^ Another study measured human and environmental microbiota in intervention studies to illustrate the role of biodiversity in enhancing immunoregulatory pathways, thereby reducing the risk of immune-mediated diseases in children.^24^ Additionally, indicators across the human, animal, and plant spectrum can be used to forecast disease risk.^25^

Cataloguing existing resources, such as inventories and assets, and increasing their accessibility and use through open access platforms are other opportunities for alignment. Data gaps can be addressed by developing cross-sector data collection infrastructures that consider differences in scale, resolution, and timeframes, including long-term monitoring of outcomes, and by establishing robust monitoring and evaluation frameworks to track progress across shared objectives. A notable example is the global linkage of biodiversity and human disease data under-taken by the Global Biodiversity Information Facility in collaboration with the World Health Special Programme on Research and Training in Diseases of Poverty.^26^ This initiative fosters open access to data as a means of supporting the early detection, monitoring, and control of vector-borne diseases.^26^

Finally, there should be an aim to enhance assessments of potential needs and effects across ecosystems and health. Environmental and social impact assessments, health impact and risk assessments, and joint strategic needs assessments (JSNAs) are long-standing best practices across both sectors. In the UK, momentum is growing for local authorities to develop JSNAs that address concerns related to climate change as well as public health and equity.^27,28^ The UK Health Security Agency’s review of climate change and public health indicators^29^ is a useful guide for JSNA development, identifying key environmental and public health indicators to monitor progress on climate action. A similar approach could be applied to enhance ecological considerations in local health assessments, supporting the evaluation of ecosystem impact on population health and informing the identification of suitable conservation actions that maximise societal benefits.

Recognising the effect of ecosystems on health can stimulate effective cross-sectoral integration between public health and ecology. Leveraging the combined knowledge base can strengthen our contribution to halt the breach of planetary health boundaries and safeguard the health gains of the last century. Global and national initiatives are increasingly acknowledging these interlinkages, such as the recently adopted Kunming-Montreal Global Biodiversity Framework, which provides an essential mandate and an opportunity to reverse biodiversity loss and protect nature.

Meeting these challenges requires substantial new resources. Dedicated funding for explicitly transdisciplinary approaches, including in public budgets and in global funding mechanisms such as the Pandemic Fund, the Green Climate Fund, and the Nature for Health Initiative, can advance understanding of nature–health interactions and safeguard the contribution of natural ecosystems to human health and wellbeing. The governance of these investments can further accelerate transdisciplinarity by emphasising collaboration from the development of Theories of Change to national and local implementation, alongside joint monitoring and evaluation.

## Figures and Tables

**Figure:**
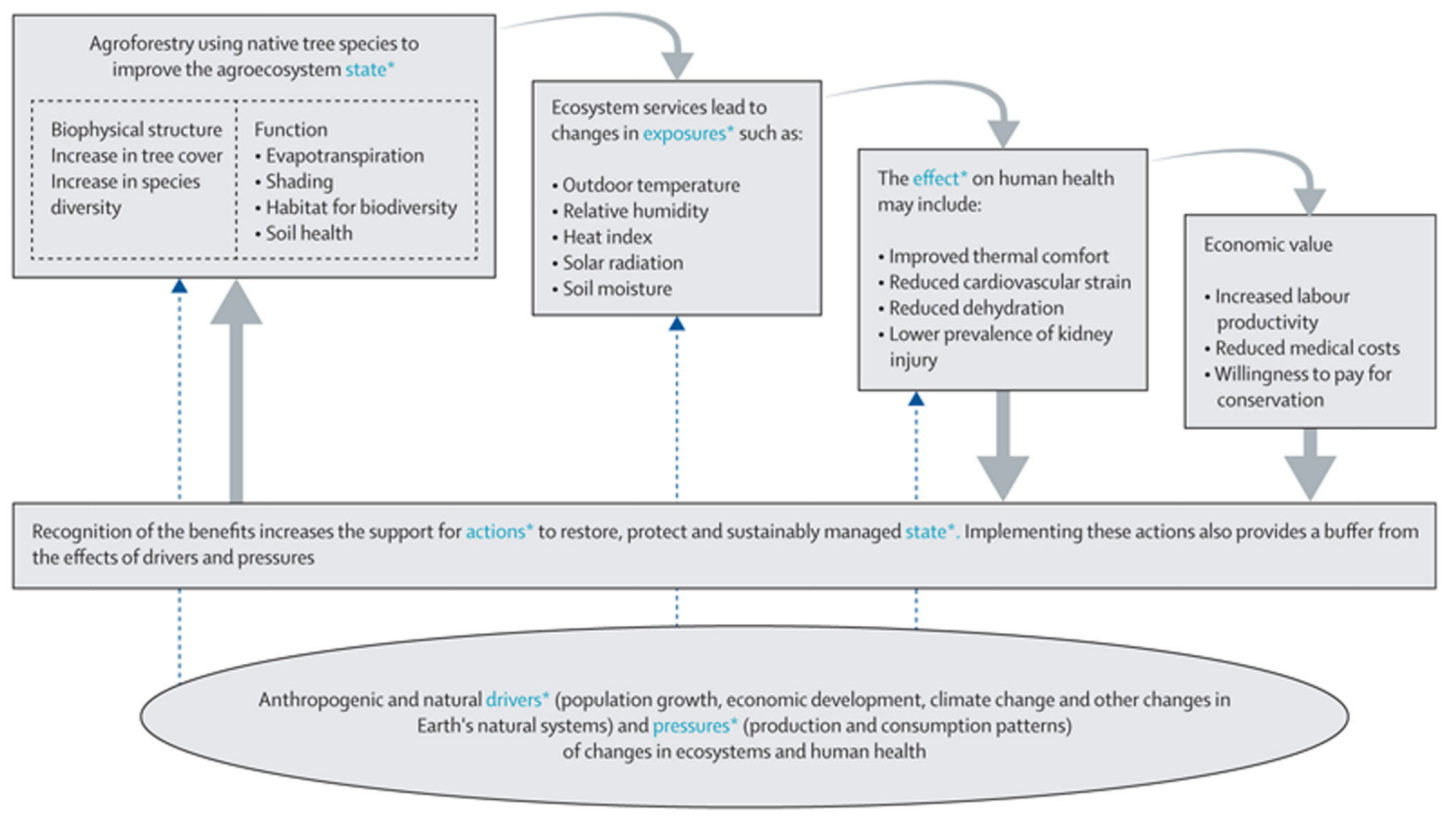
Adapted Ecosystems Cascades Model to illustrate how ecological and environmental exposure assessments can be aligned with health and economic impact analysis^19^ * indicates key components from the Driving Force-Pressure-State-Exposure-Effect-Action framework.

## *Panel:* Defining Planetary Health, One Health, and NbS

Planetary Health: The Rockefeller Foundation–*Lancet* Commission on planetary health defines Planetary Health as an approach that strives for "the achievement of the highest attainable standard of health, wellbeing, and equity worldwide through judicious attention to the human systems (political, economic, and social) that shape the future of humanity and the Earth’s natural systems that define the safe environmental limits within which humanity can flourish. Put simply, planetary health is the health of human civilisation and the state of the natural systems on which it depends".^1^

One Health: The One Health High-Level Expert Panel (OHHLEP) defines One Health as "an integrated, unifying approach that aims to sustainably balance and optimise the health of humans, animals, plants and ecosystems. One Health recognises that the health of humans, domestic and wild animals, plants, and the wider environment (including ecosystems) are closely linked and interdependent".^4^

Nature-based solutions (NbS): In 2022, the United Nations Environment Assembly (UNEA) built on previous definitions to describe NbS as "'actions to protect, conserve, restore, sustainably use, and manage natural or modified terrestrial, freshwater, coastal, and marine ecosystems which address social, economic, and environmental challenges effectively and adaptively, while simultaneously providing human wellbeing, ecosystem services, resilience, and biodiversity benefits".^5^
